# Physical activity levels are positively related to progression-free survival and reduced adverse events in advanced ER^+^ breast cancer

**DOI:** 10.1186/s12916-024-03671-x

**Published:** 2024-10-08

**Authors:** Philipp Zimmer, Tobias Esser, Diana Lueftner, Florian Schuetz, Freerk T. Baumann, Achim Rody, Andreas Schneeweiss, Andreas D. Hartkopf, Thomas Decker, Christoph Uleer, Oliver J. Stoetzer, Frank Foerster, Marcus Schmidt, Christoph Mundhenke, Karen Steindorf, Hans Tesch, Christian Jackisch, Thomas Fischer, Sven Hanson, Julia Kreuzeder, Gernot Guderian, Peter A. Fasching, Wilhelm Bloch

**Affiliations:** 1 Institute for Sport and Sport Science, Division of Performance and Health (Sports Medicine), TU Dortmund University, Dortmund, Germany; 2 Dpt. for Molecular and Cellular Sports Medicine, German Sport University Cologne, Cologne, Germany; 3 Immanuel Hospital Märkische Schweiz, AND Medical University of Brandenburg Theodor Fontane, BuckowRüdersdorf Bei Berlin, Germany; 4 Department of Obstetrics and Gynecology, University Hospital Heidelberg, Heidelberg, Germany; 5 Department I of Internal Medicine, Center for Integrated Oncology, University Hospital of Cologne, Cologne, Germany; 6 Department of Obstetrics and Gynecology, University Hospital Schleswig-Holstein, Lübeck, Germany; 7 National Center for Tumor Diseases (NCT), Heidelberg University Hospital and German Cancer Research Center, Heidelberg, Germany; 8 Dpt. of Women’s Health, University Hospital Tuebingen, Tuebingen, Germany; 9 Medical Center for Hematology and Oncology Ravensburg, Ravensburg, Germany; 10 Gynecologic Group Practice, Hildesheim, Germany; 11 Medical Center for Hematology and Oncology Munich, Munich, Germany; 12 Poliklinik GmbH Chemnitz, Chemnitz, Germany; 13 Dpt. of Obstetrics and Gynecology, Dpt. of Conservative and Molecular Gynecological Oncology, University Medical Center Mainz, Mainz, Germany; 14 Department of Obstetrics and Gynecology, Bayreuth Hospital, Bayreuth, Germany; 15 German Cancer Research Center, Division of Physical Activity, Prevention and Cancer, Heidelberg, Germany; 16 Center for Hematology and Oncology Bethanien, Frankfurt, Germany; 17 Dpt. of Obstetrics and Gynecology, Sana Klinikum Offenbach, Offenbach, Germany; 18 Winicker Norimed GmbH Medical Research, Nuernberg, Germany; 19 Novartis Pharma GmbH, Nuernberg, Germany; 20 Department of Gynecology and Obstetrics, University Hospital Erlangen, Erlangen, Germany

**Keywords:** Physical activity, Exercise, Breast cancer, Adverse events, Survival, Fatigue, Quality of life

## Abstract

**Background:**

Increased levels of physical activity are associated with a reduction of breast cancer mortality, especially in postmenopausal women with positive hormone receptor status. So far, previous observational case–control and cohort studies have focused on associations between overall leisure time physical activity and survival of women with breast cancer in general.

**Methods:**

In this multicenter prospective cohort study, conducted in Germany between 30th August 2012 to 29th December 2017, we investigated general physical activity in a homogenous sample of *n* = 1440 postmenopausal women with advanced (inoperable locally advanced or metastatic), hormone receptor-positive breast cancer receiving the same therapy (everolimus and exemestane). Self-reported physical activity was assessed using the Godin Leisure Time Exercise Questionnaire (GLTEQ) before and every 3 months during treatment. Participants were then classified into “active” and “insufficiently active” to screen their activity behavior the week prior to medical treatment. In addition, changes in physical activity patterns were assessed. Adjusted Cox regression analyses were performed for the activity categories to determine hazard ratios (HR). Besides progression-free survival (PFS), adverse events (AEs), QoL, and fatigue were assessed every 3 months until study termination.

**Results:**

Compared to “insufficiently active” patients, “active” individuals indicated a significantly longer PFS (HR: 0.84 [0.74; 0.984], *p* = .0295). No significant differences were observed for changes of physical activity behavior. Patients who reported to be “active” at baseline revealed significantly fewer AEs compared to “insufficiently” active patients. In detail, both severe and non-severe AEs occurred less frequently in the “active” patients group. In line with that, QoL and fatigue were better in physical “active” patients compared to their insufficient active counterparts at the last post-baseline assessment. Participants who remained or become active indicated less AEs, a higher QoL, and reduced fatigue levels.

**Conclusions:**

Physical activity behavior prior to medical treatment might have prognostic value in patients with advanced breast cancer in terms of extending the PFS. Moreover, physical activity before and during treatment may reduce treatment-related side effects and improve patients’ QoL and fatigue.

**Trial registration:**

EUPAS9462. Registered 30th October 2012 “retrospectively registered.”

**Supplementary Information:**

The online version contains supplementary material available at 10.1186/s12916-024-03671-x.

## Background

Besides other lifestyle factors, increased levels of physical activity are known to decrease the risk for various cancers[1]. Results from large epidemiological investigations indicate that higher levels of pre- and post-diagnosis physical activity and fitness are further associated with reduced cancer-specific and overall mortality in various types of cancer, including breast cancer[1–3]. A milestone in this context was published by Holmes et al. reporting that mortality risk was reduced by almost 50% in active patients with breast cancer compared to those who reported to be inactive(4). In addition, exercise interventions have proven to counteract several side effects of breast cancer and its medical treatment, such as fatigue, and to improve patients’ quality of life (QoL)(5–8).

To date, most epidemiological studies focusing on physical activity levels and breast cancer (progression-free) survival/mortality included more or less heterogeneous populations with different kinds of tumors and medical treatments[9]. Overall, a meta-analysis by Zhong et al. suggests that the positive influence of increased levels of physical activity on breast cancer mortality is most pronounced in postmenopausal women(10). Moreover, studies report larger effects in women with a body mass index (BMI) > 25 and in women with a positive hormone receptor status(10).

The establishment of adjuvant endocrine therapies in patients with advanced hormone receptor-positive (HR +) breast cancer is of fundamental importance. In addition to vasomotric restriction, some of the most common side effects of endocrine treatments include musculoskeletal symptoms, which are often underestimated[11]. According to Condorelli an Vaz-Luis, this can lead to poor treatment adherence and poor oncologic outcomes[11]. In the case of resistance to endocrine therapies, combining targeted pathway-based therapies and endocrine therapies is a promising therapeutic approach.

Despite the clinical establishment of combined therapies, the knowledge about the influence of physical activity on advanced breast cancer (inoperable locally advanced or metastatic) related outcomes (survival, adverse events, QoL) is still sparse. Moreover, studies in the field have tried to differentiate between different activity durations and intensities[2].

In this context, associations between self-reported physical activity levels, progression-free survival (PFS), the occurrence of adverse events, and QoL were investigated within the *BR*east cancer treatment with *A*finitor® (everolimus) and exemestane for ER + *WO*men (BRAWO) study in postmenopausal, hormone receptor-positive patients with advanced breast cancer during treatment with everolimus and exemestane.

## Methods

The multicenter prospective cohort study BRAWO was conducted in Germany between 30th August 2012 to 29th December 2017. A total of 2100 patients were enrolled across 341 centers. Observation ended 1 year after the last patient was enrolled. Out of these 2100 patients, 2074 were evaluable for efficacy and safety analysis which are reported elsewhere[12]. A total of 1440 patients had fully completed assessments of quality of life and physical activity and are included in this analysis. The study was in accordance with the Declaration of Helsinki and approved by all local ethics committees of the 341 recruiting sites (hospitals and medical practices). The study was registered at the European Union electronic Register of Post-Authorisation Studies (Trial number: EUPAS9462). Written consent was obtained by all study participants prior to inclusion.

Inclusion criteria were defined as follows: Postmenopausal women (≥ 18 years) with advanced positive hormone-receptor status (HR +) (either estrogen receptor (ER +) or progesterone receptor (PR +) or both), negative epidermal growth factor receptor-2 status (HER2 −) breast cancer without symptomatic visceral metastases, who were treated with Everolimus in combination with Exemestane, after they relapsed or progressed on non-steroidal aromatase inhibitor (e.g., anastrozole, letrozole). As defined by the 6th and 7th International Consensus guidelines for the management of advanced breast cancer (ABC guidelines 6 and 7), the disease refers to inoperable locally advanced or metastatic breast cancer[13].

All outcome measures described below were assessed after recruitment and before starting medical treatment as well as every 3 months thereafter until the end of treatment or study discontinuation.

Detailed information on patients’ characteristics is shown in Table 1 and Supplement 1.

**Table 1:** Patient characteristics. Data are presented as mean ± SD and as absolute numbers and percentages of category

	Godin: active (*N* = 332)	Godin: insufficiently active (*N* = 1108)	Total (*N* = 1440)
Age (mean ± SD, min–max, median)	63.3 ± 9.7 (36–88, 63)	65.5 ± 10.4 (20–91, 66)	65 ± 10.2 (20–91, 66)
BMI (mean ± SD, min–max, median)	26.2 ± 4.7 (17.6–46.8, 25.2)	26.7 ± 5.1 (14.4–54.4, 26)	26.6 ± 5 (14.4–54.4, 25.8)
Time since diagnosis (years) (mean ± SD, min–max, median)	9.0 ± 6.9 (0.2–39.1, 7.0)	8.8 ± 6.9 (0.2–40.4, 7.0)	8.9 ± 6.9 (0.2–40.4, 7.0)
TNM classification of the tumor at time of primary diagnosis—T, ***n*** (%)			
X	9 (2.8)	66 (6.0)	75 (5.3)
1	95 (29.1)	316 (28.7)	411 (28.8)
2	155 (47.4)	450 (40.9)	605 (42.4)
3	38 (11.6)	121 (11.0)	159 (11.1)
4	30 (9.2)	148 (13.4)	178 (12.5)
Missing	5	7	12
TNM tumor stadium at time of primary diagnosis—N, ***n*** (%)			
X	12 (3.7)	101 (9.2)	113 (7.9)
0	103 (31.4)	297 (27.0)	400 (28.0)
1	111 (33.8)	384 (34.9)	495 (34.6)
2	54 (16.5)	169 (15.3)	223 (15.6)
3	48 (14.6)	150 (13.6)	198 (13.9)
Missing	4	7	11
TNM tumor stadium at time of primary diagnosis—M, ***n*** (%)			
X	26 (8.0)	91 (8.3)	117 (8.2)
0	224 (68.7)	717 (65.2)	941 (66.0)
1	76 (23.3)	292 (26.5)	368 (25.8)
Missing	6	8	14
Grading, ***n*** (%)			
G1	16 (5.1)	50 (4.8)	66 (4.8)
G2	222 (70.5)	694 (66.2)	916 (67.2)
G3	75 (23.8)	303 (28.9)	378 (27.7)
G4	2 (0.6)	2 (0.2)	4 (0.3)
Missing	17	59	76
Histological subtype, ***n*** (%)			
Invasive ductal	215 (67.2)	744 (69.7)	959 (69.1)
Invasive lobular	65 (20.3)	229 (21.5)	294 (21.2)
Other subtypes	40 (12.5)	94 (8.8)	134 (9.7)
Missing	12	41	53
Estrogen receptor, ***n*** (%)			
Negative	7 (2.1)	23 (2.1)	30 (2.1)
Positive	324 (97.9)	1084 (97.9)	1408 (97.9)
Missing	1	1	2
Progesterone receptor, ***n*** (%)			
Negative	79 (23.9)	255 (23.0)	334 (23.2)
Positive	249 (75.2)	839 (75.8)	1088 (75.7)
Unknown	3 (0.9)	13 (1.2)	16 (1.1)
Missing	1	1	2
Metastases at primary diagnosis, ***n*** (%)			
No	270 (81.8)	869 (78.9)	1139 (79.5)
Yes	60 (18.2)	233 (21.1)	293 (20.5)
Missing	2	6	8
Metastases localization, ***n*** (%)			
Visceral metastases (lung, liver, CNS)	130 (39.4)	432 (39.1)	562 (39.1)
Visceral and bone metastases	65 (19.7)	278 (25.1)	343 (23.9)
Visceral without bone metastases	65 (19.7)	154 (13.9)	219 (15.3)
Therapy line, ***n*** (%)			
1st line	98 (29.5)	310 (28.0)	408 (28.3)
2nd line	111 (33.4)	339 (30.6)	450 (31.3)
3rd line	58 (17.5)	217 (19.6)	275 (19.1)
4th line	32 (9.6)	122 (11.0)	154 (10.7)
5th line (and later)	33 (9.9)	120 (10.8)	153 (10.6)
Prior antineoplastic surgery, ***n*** (%)	309 (93.1)	1011 (91.2)	1320 (91.7)
Prior antineoplastic radiation, ***n*** (%)	278 (83.7)	897 (81.0)	1175 (81.6)

### Outcome measures

Patients were invited to complete the Godin Leisure Time Exercise Questionnaire (GLTEQ), to assess their overall physical activity level in everyday life. The Godin-Leisure-Time Exercise Questionnaire has shown good reliability and validity. The internal consistency (Cronbach's alpha) ranges from 0.80 to 0.85. The test–retest reliability ranges from 0.81 to 0.91. In terms of validity, the questionnaire has moderate to strong correlations with other established methods for measuring physical activity, such as physiological measurements (heart rate, VO2max) and other self-reported questionnaires[14–16]. Patients are asked to indicate the number of physical activities lasting more than 15 min in three graded categories (strenuous exercise, moderate exercise, light exercise). Examples of each category were provided. Prior briefing was given in the clinical context regarding the three categories subsequently used for evaluation. Based on the reported amount, the leisure-time score index (LSI) was calculated by multiplying the corresponding metabolic equivalents ((frequency of moderate × 5) + (frequency of strenuous × 9)). Following Godin, only strenuous and moderate activities were included for classification and calculation of LSI[14]. The cut-point for the LSI is often used to divide participants into groups with sufficient and insufficient physical activity. A common threshold is a LSI of 24 units or more per week. This threshold represents a minimum amount of physical activity recommended by the World Health Organization (WHO) for health benefits(17). LSI levels of 24 or more are therefore comparable to physical activity levels which consist of 150 min of moderate-intensity physical activity per week, or 75 min of strenuous physical activity per week, or an equivalent combination of both intensities. Further categories are (i) 14 to 23 units: moderately active (some health benefits) and (ii) less than 14 units: insufficiently active (less substantial or low benefits). Thus, individuals with an LSI ≤ 23 were classified as insufficiently active, and individuals with an LSI ≥ 24 were classified as active.

PFS was evaluated and documented by the responsible physician at each recruiting site. PFS was defined as the time from the date of baseline visit + 1 to disease progression or death. If the day or the month was missing in the date of progression or death, this was replaced by “1.” Patients were followed up until progression or death and were only censored due to lost-to-follow-up or survival without progression or death at the official end of study. The documentation of the end of treatment with Everolimus (for any reason) was carried out at the soonest possible time that is in line with clinical practice. PFS was followed up if treatment discontinuation occurred for any other reason than progression. Overall, survival was not followed up separately in this study. However, PFS includes disease progression as well as death.

Adverse events (AEs), serious AEs (Grades 3 and 4) and non-serious AEs (stomatitis, nausea, diarrhea, etc., Grades 1 and 2), QoL and fatigue (EORTC QLQ-C30 questionnaires[18] were recorded at baseline and were repeated every 3 months until the end of treatment or study discontinuation. Grading of AEs was based on the Common Terminology Criteria for Adverse Events (CTCAE). A detailed overview can be found in the primary endpoint paper of this study[12].

### Statistical analyses

All statistical analyses were completed using SAS software version 9.2. In a first step descriptive statistics (sample statistics, frequency tables) were calculated for all parameters. In case parameters were observed repeatedly, the statistics were analyzed by visit. Regarding PFS, analyses were performed univariately in a first step calculating Kaplan–Meier estimates and presenting Kaplan–Meier curves in total and for selected subgroups. Following this Cox regression analyses were conducted for complete cases to determine hazard ratios (HR) for the two exercise categories adjusting for the following prognostic factors/covariates: age [≤ 65 years; 66–74 years, > 75 years], body mass index (BMI) [< 20, 20–25, 26–29, > 29], duration from diagnosis to first metastasis [0–2 years, > 2–5 years, > 5 years], tumor grading [G1, G2, G3 + G4], therapy line [1st line, 2nd/3rd line, 4th and higher] and occurrence of visceral metastasis [yes/no]. Results are presented as *p*-values, hazard ratios, and their corresponding 95% confidence intervals (CI). Analyses were repeated including only factors/covariates that showed a significance level of at least 0.1 or were assessed as medically relevant by the medical experts.

The occurrence (yes/no) of overall adverse events (AEs), serious AEs, and non-serious AEs between the two exercise levels at baseline was compared using logistic regression models including the same covariates which have been used for PFS analysis. Finally, the last post-baseline recorded global QoL and the QoL subscale fatigue values were compared between the two activity levels at baseline using ANCOVA models.

All analyses described above were also conducted for changes in physical activity behavior from baseline to the last post-baseline assessment. For this purpose, four groups were built: Participants who stayed active (active/active), participants who became active (insufficiently active/active), participants who became inactive (active/insufficiently active), and participants who remained inactive (insufficiently active/insufficiently active).

Again, analyses were adjusted for all covariates mentioned above. Throughout all analyses, the level of significance was set at alpha = 0.05.

## Results

Study participants’ characteristics are shown in Table 1. Out of 2,100 enrolled patients with breast cancer, 1440 complete cases were included for analysis of PFS. In view of physical activity levels at baseline, 1108 patients were reported to be “insufficiently active” and 332 to be “active”. Median treatment duration was 4.8 months (range 1–58.0 months). In regards of changes in physical activity behavior, data from 1184 participants were available. Out of these 1184 participants, 143 (12.1%) remained physically active, 116 (10%) became active, 147 (12.4%) became insufficiently active, and 778 (65.7%) remained insufficiently active.

### PFS

**Fig. 1:**
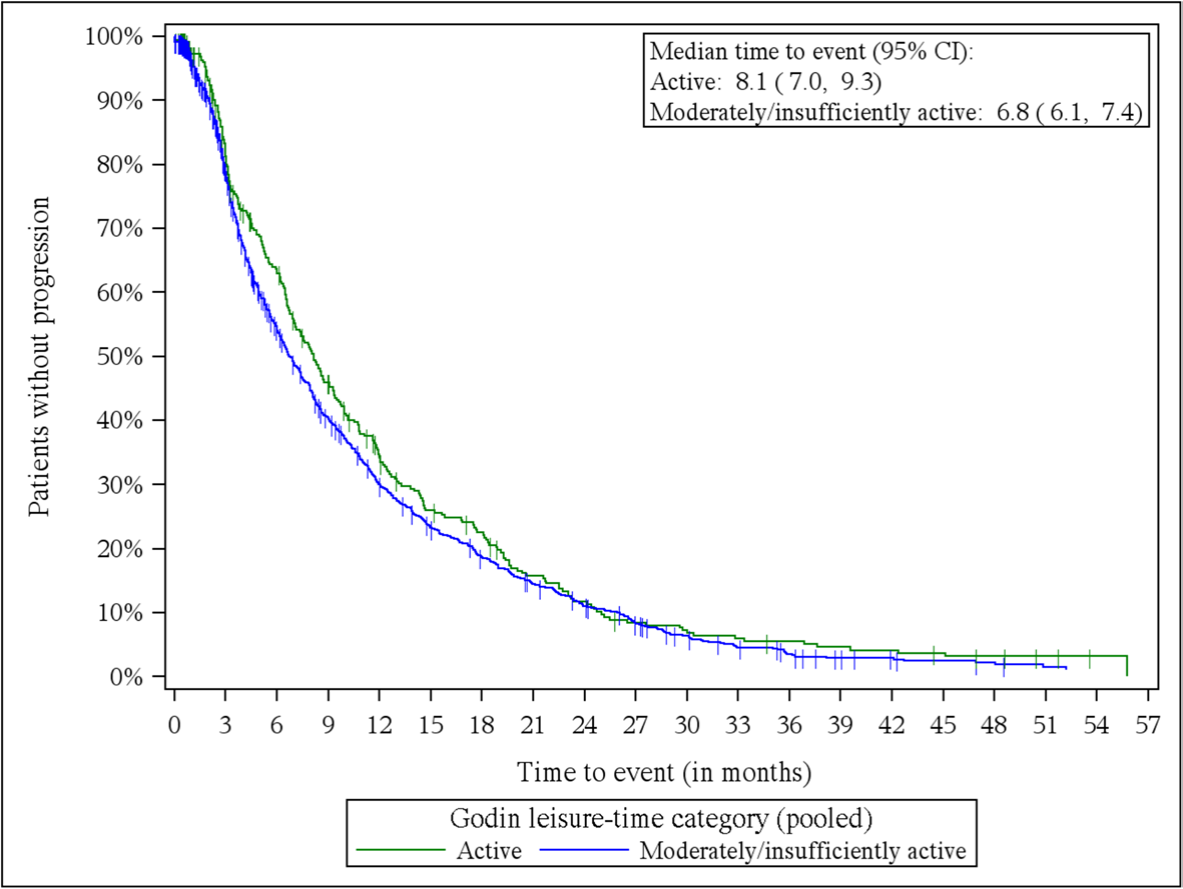
Kaplan–Meier estimates progression-free survival—by subgroups based on Godin Leisure-Time Exercise. Active: *n* = 332, insufficiently active: *n* = 1108

Kaplan–Meier estimates are shown in Fig. 1. Cox proportional hazard models (adjusted for age, BMI, tumor grading, visceral metastases (yes/no), therapy line, and duration until diagnosis of first metastases) indicated a significant effect on PFS for self-reported physical activity (*p* = 0.0295). For physical activity subgroup “active” patients (*n* = 332) revealed a significant prolonged PFS compared to those patients who reported to be “insufficiently active” (*n* = 1108) (HR: 0.84 [0.74; 0.984].).

In regards of changes in physical activity behavior results did not reach statistical significance (*p* = 0.0691): Remaining active vs. staying insufficiently active (HR: 0.897 [0.728; 1.104]), remaining active vs. becoming insufficiently active (HR: 1.12 [0.857; 1.463]), remaining active vs. becoming active (HR: 1.095 [0.830; 1.446]), becoming active vs. becoming insufficiently active (HR: 1.023 [0.775; 1.350]), becoming active vs. staying insufficiently active (HR: 0.819 [0.657; 1.020]), becoming insufficiently active vs. staying insufficiently active (HR: 0.801 [0.654; 0.981]).

### Adverse events

Logistic regression indicated a significant relation between baseline physical activity levels and the occurrence of overall AEs (*p* = 0.001). Compared to “active” patients, those who reported being “insufficiently active” active revealed an increased probability of developing AEs (OR: 2.22 [95% CI: 1.43–3.45], *p* < 0.001). Regarding serious AEs, “Insufficiently active” patients have significantly increased odds compared to “active” patients (OR: 1.40 [95% CI: 1.09–1.80]; *p* = 0.008). In view of non-serious AEs, logistic regression models indicated a significant relation as well (OR: 1.48 [95% CI: 1.04–2.12]; *p* = 0.032). Indeed, “insufficiently active” patients had more non-serious adverse events than “active” patients.

In view of changes in physical activity behavior logistic regression revealed a significant relation between activity groups and the occurrence of overall AEs (*p* < 0.001), serious AEs (*p* < 0.001), and non-serious AEs (*p* = 0.022).

Compared to patients who remained physically active those who stayed insufficiently active showed a significantly increased risk for overall AEs (*p* < 0.001; OR: 3.35 [1.85; 6.07]). Additionally, patients who became active indicated increased numbers of AEs compared to patients who remained active (*p* = 0.018; OR: 3.40 [1.23; 9.4]). All other overall AE comparisons did not reach statistical significance.

Patients who reported to stay physically active indicated significantly less serious AEs compared to those individuals who remained insufficiently active (*p* < 0.001; OR: 2.13 [1.44; 3.13]) and to those who became insufficiently active (*p* = 0.002; OR: 2.13 [1.31; 3.45]). Moreover, patients who became active indicated significantly less serious AEs compared to participants who remained insufficiently active (*p* = 0.048; OR: 0.67 [0.45; 1.0]).

**Fig. 2:**
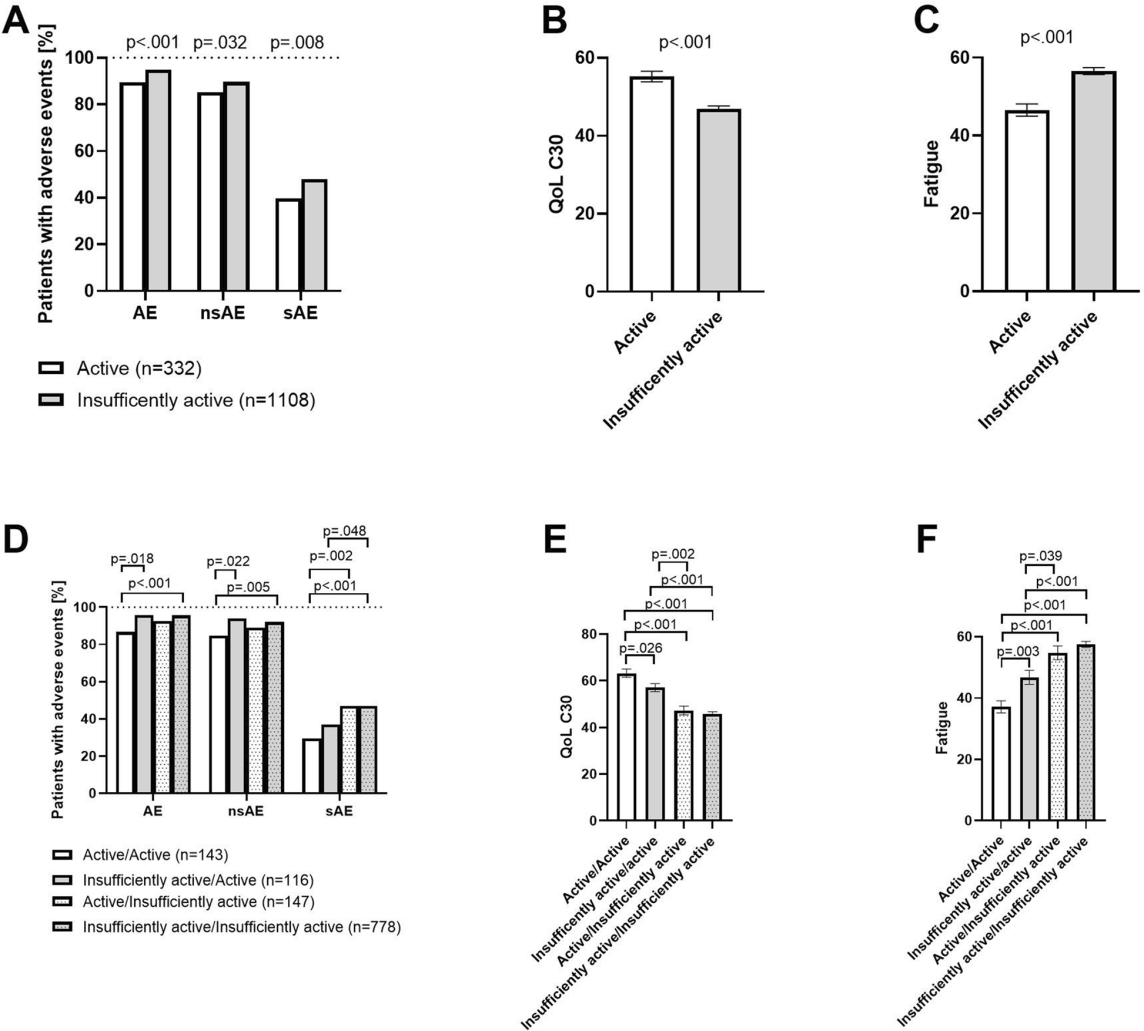
Comparison of adverse events, quality of life, and fatigue between baseline physical activity groups (**A**–**C**) as well as for changes in physical activity behavior during the course of the study (**D**–**F**). Occurrence of adverse events (AE), non-serious adverse events (nsAE), serious adverse events (sAE) (**A**), quality of life (QoL) (**B**), and fatigue (**C**) in relation to baseline physical activity groups. For **A**, the bars represent the proportion of participants who experienced at least one of the AEs shown. *P*-values for significant differences between groups in adverse events, quality of life, and fatigue are annotated

Regarding the occurrence of non-serious AEs, significant differences were found between subjects who remained active and those who remained inactive (*p* = 0.005; OR: 2.10 [1.24; 3.54]) and between those who remained active and those who became active (*p* = 0.022; OR: 2.83 [1.16; 6.89]). Results are shown in Fig. 2.

### Quality of life and fatigue

Patients who reported a more active lifestyle at baseline revealed a significantly higher QoL at the last post-baseline assessment. In detail, “active” participants showed significantly higher QoL compared to their “insufficiently active” counterparts (*p* < 0.001) (Fig. 2B).

As fatigue represents the most frequently observed side effect of anti-cancer treatment negatively affecting QoL, we also highlighted the corresponding subscales of the EORTC-QLQ-C30. Patients who reported being “active” at baseline, indicated significantly less fatigue compared to “insufficiently active” participants at the last post-baseline assessment (“active” vs. “insufficiently active”: *p* < 0.001).

ANCOVA models indicated that changes in physical activity significantly influence QoL (*p* < 0.001) and fatigue levels (*p* < 0.001). Patients who remained active revealed higher QoL and lower fatigue levels compared to patients who remained insufficiently active (*p* < 0.001; *p* < 0.001), to those who became insufficiently active (*p* < 0.001; *p* < 0.001), and to those who became active (*p* = 0.026; *p* = 0.003). Patients who reported to become active indicated significantly higher QoL and lower fatigue levels compared to patients who remained insufficiently active (*p* < 0.001; *p* < 0.001). Finally, patients who became active showed higher QoL and lower fatigue levels compared to those who became insufficiently active (*p* = 0.002; *p* = 0.039). Results are shown in Fig. 2.

## Discussion

To our knowledge, this is the first large-scale observational cohort trial investigating the influence of self-reported physical activity in daily living prior to therapy as well as during therapy on PFS, AEs, QoL, and fatigue in a homogenous population with advanced breast cancer, receiving the same type of therapy. In general, the results of this trial support findings from earlier studies reporting the beneficial effects of physical activity on PFS, AEs, QoL, and fatigue[8].

Physical activity behavior prior to medical treatment was significantly associated with PFS, whereas changes of physical activity behavior during treatment were not. These results are in line with current literature, suggesting a clear association between baseline physical activity, all-cause, and (breast) cancer-specific mortality. Studies focusing on changes of physical activity behavior showed contradictory results[19]. The latter could be explained by huge differences in study designs and methods (e.g., heterogenous populations, varying observational periods and outcomes (PFS, mortality, etc.) and assessments of physical activity)[20], and the low number of available investigations[2]. A lack of significance may further be argued with a decreased statistical power due to an increase in the numbers of groups and a slightly reduced number of observations.

Against the backdrop that all study participants received a similar medication, the BRAWO trial allows to investigate whether physical activity is associated with the occurrence of side effects*.* In fact, not only sAEs occurred more frequently in participants classified as “insufficiently active,” but also nsAEs. Of note, the results of this trial suggest that staying or becoming physically active during treatment may reduce the occurrence of overall and serious AEs. Unfortunately, whether certain AEs were induced by physical activity cannot be concluded from our data. Our data also do not allow to state on potential additional benefits of specific exercise modalities and programs in order to reduce AEs. However, a growing body of literature suggests that targeted supervised and non-supervised physical exercise programs counteracted disease- and treatment-related side effects[21–23].

In view of QoL and fatigue, the data point also to a clear positive influence in physically active people suffering from advanced breast cancer. Thus, higher levels of physical activity before medical treatment is related to increased quality of life and decreased fatigue levels at the last post-baseline assessment. Our results further support the hypothesis that keep being physically active seems to be beneficial in regards of both, QoL and fatigue. Indeed, participants who became more active during treatment also indicated higher QoL and reduced fatigue levels compared to those who became less active or remained inactive. Although previous research suggests that physical activity during adjuvant chemotherapy improves side effects, these findings are mostly limited to early-stage breast and prostate cancer[24, 25]. Given the special disease and treatment considerations in patients with advanced breast cancer, our results suggest that physical activity may also reduce AEs and side effects during treatment in the advanced stage of the disease.

The results of this trial should be interpreted within the context of its strengths and limitations. Strengths include the large sample size and the inclusion of a homogenous population in view of menopausal status, receptor status, and medical treatment. The use of self-report questionnaires, particularly the GLTEQ, is a well-established and simple attempt to assess physical activity. Specifically, the categorization into active and insufficiently active patients is not only used in the physical activity guidelines for cancer survivors[26], but also validated particularly in breast cancer patients[27].

Even though the method used allows to count for specific metabolic equivalents of task (MET)[28], it was shown, that self-reported measures of physical activity are relatively imprecise[29] and cover only a limited time interval (here: leisure time physical activity in the week prior to initiation of treatment). Therefore, calculating exact MET levels is at least questionable. Furthermore, the limitations of such activity questionnaires remain. For instance, a relatively rough memory-based assessment of activity, including duration, frequency, and intensity of activity, is made, which is open to recall errors (e.g., when the patient is asked for the exact hours or minutes spent in certain activities).

If possible, future trials may use objective activity trackers to collect more detailed information on the patients’ physical activity. Most other epidemiological studies used cancer-specific and overall mortality as primary endpoints. In contrast, we analyzed PFS. Thereby, we cannot definitely conclude that activity levels influence overall and cancer-specific survival in the investigated population. In regards of fatigue, we have used the corresponding subscale of the EORTC QLQ-C30 questionnaire due to its practicability. Following studies may use a more robust/detailed assessment focusing on this relevant side effect and include more detailed information on other lifestyle factors and habits, such as nutrition and drug consumption (e.g., alcohol, smoking).

## Conclusions

In conclusion, the results of the present investigation underline the importance of staying physically active in the course of disease—even in an advanced setting. Patients self-assessing themselves as physically active may have benefits regarding PFS, AEs, and QoL. Patients should be encouraged to stay physically active or be motivated to increase their physical activity level according to the WHO guidelines[17]. Although limited, simple questionnaires, such as the GLTEQ may help clinicians to identify potential deficits of their patients and to provide recommendations accordingly. Further prospective randomized controlled trials should investigate the supportive effects of different exercise modalities (e.g., endurance vs. strength training) and effects in therapeutic subgroups in patients with breast cancer.

## Supplementary Information

Supplementary Material 1: Supplement 1. Patient characteristics for the four subgroups according to maintenance or change in physical activity behavior. Data are presented as mean ± SD, and as absolute numbers and percentages of category.

## Data Availability

The data that support the findings of this study are available from the corresponding author, upon reasonable request.

## Abbreviations

QoL: Quality of life
BMI: Body mass index
HR +: Hormone receptor-positive
PFS: Progression-free survival
ER +: Estrogen receptor-positive
PR +: Progesterone receptor-positive
HER2 −: Negative epidermal growth factor receptor-2 status
GLTEQ: Godin Leisure Time Exercise Questionnaire
LSI: Leisure time score index
AE: Adverse events
HR: Hazard ratios
MET: Metabolic equivalents of task
