# Supplementary material for: Physical activity levels are positively related to progression-free survival and reduced adverse events in advanced ER+ breast cancer
Source: BMC Med. 2024 Oct 8;22:442. doi: 10.1186/s12916-024-03671-x (PMC11462731; doi:10.1186/s12916-024-03671-x)
Supplement: Supplementary file 1 — Supplementary Material 1: Supplement 1. Patient characteristics for the four subgroups according to maintenance or change in physical activity behavior. Data are presented as mean ± SD, and as absolute numbers and percentages of category. [file 12916_2024_3671_MOESM1_ESM.docx]

**Suppplement 1:** Patient characteristics for the four subgroups according to maintenance or change in physical activity behavior. Data are presented as mean ± SD, and as absolute numbers and percentages of category.

|  | Godin: Active / Active  (N=143) | Godin Insufficiently active / Active (N=116) | Godin Active / Insufficiently active (N=147) | Godin: Insufficiently active / Insufficiently active (N=778) |
| --- | --- | --- | --- | --- |
| **Age (mean±SD, min-max, median)** | 61.1 ± 9.2 (41 – 88, 61) | 63.0 ± 9.4 (26 – 90, 64) | 64.7 ± 9.7 (36 – 87, 65) | 65.1 ± 10.5 (20 – 91, 66) |
| **BMI (mean±SD, min-max, median)** | 25.6 ± 4.2 (17.6 – 37.1, 24.8) | 25.6 ± 4.1 (17.9 – 41.5, 25.0) | 26.6 ± 5.1 (18.6 – 46.8, 25.6) | 27.0 ± 5.2 (14.4 – 54.4, 26.4) |
| **Time since diagnosis (years) (mean±SD, min-max, median)** | 8.2 ± 6.4 (0.3 – 30.9, 6.1) | 8.7 ± 6.6 (0.3 – 30.3, 6.5) | 9.7 ± 7.5 (0.2 – 39.1, 7.8) | 8.7 ± 6.7 (0.2 – 37.4, 6.9) |
| **TNM classification of the tumour at time of primary diagnosis - T, n (%)** |  |  |  |  |
| X | 5 (3.5) | 7 (6.0) | 3 (2.1) | 41 (5.3) |
| 1 | 41 (28.9) | 36 (31.0) | 43 (29.7) | 222 (28.7) |
| 2 | 65 (45.8) | 55 (47.4) | 70 (48.3) | 312 (40.4) |
| 3 | 18 (12.7) | 9 (7.8) | 18 (12.4) | 93 (12.0) |
| 4 | 13 (9.2) | 9 (7.8) | 11 (7.6) | 105 (13.6) |
| Missing | 1 | 0 | 2 | 5 |
| **TNM tumor stadium at time of primary diagnosis - N, n (%)** |  |  |  |  |
| X | 3 (2.1) | 7 (6.0) | 7 (4.8) | 64 (8.3) |
| 0 | 39 (27.5) | 36 (31.0) | 51 (34.9) | 213 (27.6) |
| 1 | 56 (39.4) | 40 (34.5) | 44 (30.1) | 263 (34.0) |
| 2 | 24 (16.9) | 17 (14.7) | 21 (14.4) | 122 (15.8) |
| 3 | 20 (14.1) | 16 (13.8) | 23 (15.8) | 111 (14.4) |
| Missing | 1 | 0 | 1 | 5 |
| **TNM tumor stadium at time of primary diagnosis - M, n (%)** |  |  |  |  |
| X | 11 (7.8) | 7 (6.0) | 12 (8.3) | 60 (7.8) |
| 0 | 90 (63.4) | 87 (75.0) | 105 (72.9) | 499 (64.6) |
| 1 | 41 (28.9) | 22 (19.0) | 27 (18.8) | 213 (27.6) |
| Missing | 1 | 0 | 3 | 6 |
| **Grading, n (%)** |  |  |  |  |
| G1 | 9 (6.7) | 6 (5.3) | 7 (4.9) | 33 (4.5) |
| G2 | 86 (63.7) | 69 (61.1) | 105 (73.9) | 502 (68.1) |
| G3 | 39 (28.9) | 38 (33.6) | 29 (20.4) | 200 (27.1) |
| G4 | 1 (0.7) | 0 (0.0) | 1 (0.7) | 2 (0.3) |
| Missing | 8 | 3 | 5 | 51 |
| **Histological subtype, n (%)** |  |  |  |  |
| Invasive ductal | 90 (64.8) | 84 (73.7) | 95 (67.9) | 524 (70.0) |
| Invasive lobular | 33 (23.7) | 20 (17.5) | 27 (19.3) | 163 (21.8) |
| Other subtype | 16 (11.5) | 10 (8.8) | 18 (12.9) | 62 (8.3) |
| Missing | 4 | 2 | 7 | 29 |
| **Estrogen receptor, n (%)** |  |  |  |  |
| Negative | 4 (2.8) | 4 (3.5) | 3 (2.0) | 16 (2.1) |
| Positive | 138 (97.2) | 112 (96.6) | 144 (98.0) | 762 (98.0) |
| Missing | 1 | 0 | 0 | 0 |
| **Progesterone receptor, n (%)** |  |  |  |  |
| Negative | 33 (23.2) | 25 (21.6) | 35 (23.8) | 180 (23.1) |
| Positive | 107 (75.4) | 89 (76.7) | 111 (75.5) | 592 (76.1) |
| Unknown | 2 (1.4) | 2 (1.7) | 1 (0.7) | 6 (0.8) |
| Missing | 1 | 0 | 0 | 0 |
| **Metastases at primary diagnosis, n (%)** |  |  |  |  |
| No | 112 (78.9) | 97 (83.6) | 124 (84.9) | 609 (78.6) |
| Yes | 30 (21.1) | 19 (16.4) | 22 (15.1) | 166 (21.4) |
| Missing | 1 | 0 | 1 | 3 |
| **Metastases localization, n (%)** |  |  |  |  |
| Visceral metastases (lung, liver, CNS) | 62 (43.7) | 47 (40.5) | 52 (35.6) | 305 (39.3) |
| Visceral and bone metastases | 36 (25.4) | 26 (22.4) | 21 (14.4) | 199 (25.6) |
| Visceral without bone metastases | 26 (18.3) | 21 (18.1) | 31 (21.2) | 106 (13.6) |
| **Therapy line, n (%)** |  |  |  |  |
| 1st line | 37 (25.9) | 37 (31.9) | 49 (33.3) | 223 (28.7) |
| 2nd line | 44 (30.8) | 34 (29.3) | 49 (33.3) | 236 (30.3) |
| 3rd line | 26 (18.2) | 18 (15.5) | 27 (18.4) | 157 (20.2) |
| 4th line | 17 (11.9) | 9 (7.8) | 9 (6.1) | 89 (11.4) |
| 5th line (and later) | 19 (13.3) | 18 (15.5) | 13 (8.8) | 73 (9.4) |
| **Prior antineoplastic surgery, n (%)** | 131 (91.6) | 107 (92.2) | 140 (95.2) | 714 (91.8) |
| **Prior antineoplastic radiation, n (%)** | 121 (84.6) | 101 (87.1) | 124 (84.4) | 622 (80.0) |
